# Genetic Association Between Growth Differentiation Factor 5 Single Nucleotide Polymorphism and Primary Knee Osteoarthritis in a Group of Egyptian Patients: A Pilot Study

**DOI:** 10.31138/mjr.30.2.114

**Published:** 2019-06-29

**Authors:** Dia Mohamed Fahmy Mohasseb, Emmanuel Kamal Aziz Saba, Neveen Lewis Mikhael Saad, Amira Dimas Hanna Sarofeem

**Affiliations:** 1 Physical Medicine, Rheumatology and Rehabilitation Department, Faculty of Medicine, Alexandria University, Egypt,; 2 Clinical and Chemical Pathology Department, Faculty of Medicine, Alexandria University, Egypt,; 3 Physical Medicine, Rheumatology and Rehabilitation Department, Ministry of Health, Alexandria Governorate, Egypt

**Keywords:** Knee osteoarthritis, Growth Differentiation Factor 5, GDF5 polymorphism, single nucleotide polymorphism, allelic discrimination

## Abstract

**Aim:**

This study aimed to determine the genetic association between Growth Differentiation Factor 5 (GDF5) gene (rs143383 T/C) single nucleotide polymorphism (SNP) and primary knee osteoarthritis (OA) in a group of Egyptian patients.

**Patients and Methods:**

The study included 47 patients with primary knee OA and 40 apparently healthy control subjects. The disease was assessed using Western Ontario and McMaster Universities Osteoarthritis Index (WOMAC) score and Health Assessment Questionnaire (HAQ). Radiological assessment was done by Kellgren-Laurence (K/L) grading system. The genetic association of the SNP with primary knee OA was assessed by restriction fragment length polymorphism - polymerase chain reaction (RFLP-PCR).

**Results:**

The mean total WOMAC index was significantly higher in patients with TT genotype as compared to patients with CC and CT genotypes (*P*<0.001). Similarly, the HAQ score was significantly higher among patients with TT genotype when compared to patients with CT and CC genotypes (*P*<0.001). There was a statistically significant association between different GDF5 genotypes and K/L radiological grading of knee OA among the studied patients (*P*=0.029). No statistically significant association was detected on comparing the frequency distribution of GDF5 alleles and genotypes frequencies of the SNP in patients and healthy controls.

**Conclusion:**

There is a possible genetic association between GDF5 (rs143383) SNP and severity of primary knee OA, which might facilitate the detection of patients with high risk for disease progression. The present study did not detect an association between the SNP and development of primary knee OA.

## INTRODUCTION

Osteoarthritis (OA) is the most common chronic, degenerative, and disabling joint disease worldwide.^1^ Primary knee OA is the most common form of OA which commonly affects individuals over 45 years of age.^2,3^ The major clinical manifestations of knee OA are pain and stiffness. Knee OA leads to physical and psychosocial disability associated with deterioration of quality of life.^4,5^

Osteoarthritis is a multifactorial joint disease. Many metabolic, biochemical and genetic factors are among the major risk factors associated with the onset and development of OA.^6^ Heritability studies have shown that genetic components account for approximately half of the risk for development of primary knee OA.^7^ In addition, various genetic polymorphisms may be associated with knee OA in certain ethnic groups.^8^

The most important OA risk allele has been found to be Growth Differentiation Factor 5 (GDF5) (rs143383), a C/T single nucleotide polymorphism (SNP).^8–11^ The GDF5 gene encodes the expression of GDF5 protein. GDF5 is also known as cartilage-derived morphogenetic protein 1. It is a member of the transforming growth factor-β superfamily.^10^ It participates in the development, maintenance, and repair of different tissues in the synovial joint, including bone, cartilage and other soft tissues present in the synovial joint.^11^ Severe rare mutations of the GDF5 gene exist, that result in dominant musculoskeletal defects and deformities.^9,10^

This study aimed to determine the genetic association between the GDF5 gene (rs143383 T/C) SNP and primary knee OA in a group of Egyptian patients.

## PATIENTS AND METHODS

### Patients

The current study included 47 primary knee OA patients. All included patients fulfilled the American College of Rheumatology criteria for classification of primary knee OA.^12^ The patients were recruited sequentially from those attending the outpatient clinic of Physical Medicine, Rheumatology and Rehabilitation Department, Main University Hospital, Alexandria Faculty of Medicine, Egypt. A control group of 40 apparently healthy volunteers were included. The volunteers consisted of medical staff, their relatives and patients^’^ relatives. Patients diagnosed as secondary OA were excluded from the study.

The study was explained to the participants, and an informed consent was given by each. The study had been approved by the Ethics Committee of the Faculty of Medicine, Alexandria University, Egypt.

### Methods

**Figure 1.**
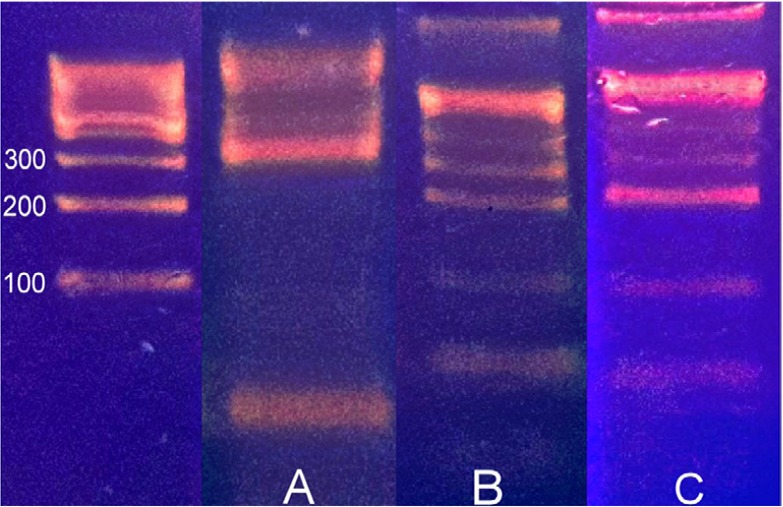
This figure shows the bands of each Growth Differentiation Factor 5 genotype in the electrophoresis gel: A represents TT (fragment length 344 bp), B represents TC (fragment length 104, 230, and 344) and C represents CC (fragment length 104, 230).

The patients included in the study were subjected to full history taking and clinical examination of both knees. Anthropometric measurements (weight, height, body mass index [BMI] [kg/m^2^]) were measured.^13^ The severity of knee OA symptoms was assessed using the Western Ontario and McMaster Universities Osteoarthritis Index (WOMAC).^14^ Functional assessment of the patients was done by using Health Assessment Questionnaire (HAQ).^15^ The severity of knee OA was scaled radiographically by using the Kellgren-Laurence (K/L) grading system. The severity was categorized as follows: very mild (grade 1), mild (grade 2), moderate (grade 3) and severe (grade 4).^16^ GDF5 (rs143383) SNP was detected by restriction fragment length polymorphism-polymerase chain reaction (RFLP-PCR). Amplification of the promoter area of GDF5 gene was done using PCR primers with primer sequence GATTTTTTCTGAGCACCTGCAGG (forward) and GTGTGTGTTTGTATCCAG (reverse) (Applied Biosystems). 50 μl PCR mixture contained 100 ng of genomic DNA, 20 pmol of each primer, 10 μl of master mix and 1 unit of Taq DNA polymerase (Red DNA Polymerase-USA). The PCR reaction was started with an initial denaturation at 95°C for 5 min, followed by 35 cycles of amplification in a thermocycler (Veriti) with denaturation at 94°C for 1 min, annealing at 58°C for 1 min, extension at 72°C for 1 min, and final extension at 72°C for 10 min. 10 μL of PCR product was incubated at 37°C with 3 units of BsiEI for 4 hours. The digested product was electrophoresed on 2% agarose gel with ethidium bromide staining before being visualized on a UV transilluminator (figure 1).^17^

#### Statistical analysis

Data were fed to the computer and analyzed using IBM SPSS software package version 20.0. (Armonk, NY: IBM Corp).^18^ The Kolmogorov-Smirnov test was used to verify the normality of distribution of variables. Comparisons between groups for categorical variables were assessed using Chi-square test (Fisher or Monte Carlo). Student t-test was used to compare two groups for normally distributed quantitative variables. Kruskal-Wallis test was used to compare different groups for not normally distributed quantitative variables and followed by Post Hoc test (Dunn’s) for pair wise comparison. Odd ratio (OR) and 95% Confidence Interval were assessed. Significance of the obtained results was judged at the 5% level.

## RESULTS

The present study included 47 patients with primary knee OA (40 women [85.1%] and 7 men [14.9%]). Their mean age was 54.13 ± 8.46 years (ranged from 40 to 76 years). The control group consisted of 40 apparently healthy individuals (29 women [72.5%] and 11 men [27.5%]). Their mean age was 51.30 ± 8.67 years (ranged from 40 to 64 years). There were no statistically significant differences between patients and control group as regards gender (X^2^=2.093, *P*=0.148) and age (t=1.131, *P*=0.681). WOMAC of moderate degree was the commonest grade constituted 51.1%. HAQ of mild-moderate disability was the most common grade and constituted 51.1%. Kellgren-Lawrence grade 3, the most common grade, constituted 51.1% for the right knee and 46.9% for the left knee. The demographic, anthropometric, clinical and radiological characteristics of patients and control subjects are summarized in *Table 1*.

**Table 1.** Demographic, anthropometric, clinical and radiological characteristics of patients and control subjects.

**Demographic, anthropometric, clinical and radiological characteristics**	**Patients (n=47 patients)**	**Control subjects (n=40 subjects)**	**Test of significance**	***P***
Women (number [percentage], subjects)	40 (85.1%)	29 (72.5%)	(X^2^) 2.093	0.148^†^
Age (mean±SD, years)	54.13 ± 8.46	52.03 ± 8.86	(t) 1.131	0.261
Weight (mean±SD, kg)	82.21 ± 15.34	79.38 ± 13.71	(t) 0.903	0.369
Height (mean±SD, cm)	159.13 ± 7.25	161.05 ± 5.78	(t) 1.350	0.181
BMI (mean±SD, kg/m^2^)	32.50 ± 6.02	30.47 ± 3.98	(t) 1.816	0.073
BMI grading [number (percentage), subjects]				
Overweight (25-<30)	19 (40.4%)	20 (50.0%)	(X^2^) 1.720	0.476
Obese (30-<40)	24 (51.1%)	19 (47.5%)		
Morbid obesity (≥40)	4 (8.5%)	1 (2.5%)		
Laterality (unilateral/bilateral) [number (percentage), subjects]	4 (8.5%)/43(91.5%)	NA	NA	NA
Duration of the condition (mean±SD, years)	3.29 ± 2.83	NA	NA	NA
WOMAC (mean±SD)	47.66 ± 13.42	NA	NA	NA
Pain subscale (mean±SD)	9.68 ± 2.84	NA	NA	NA
Stiffness subscale (mean±SD)	2.30 ± 1.55	NA	NA	NA
Function difficulty subscale (mean±SD)	35.68 ± 9.88	NA	NA	NA
HAQ score (mean±SD)	0.97 ± 0.31	NA	NA	NA
Kellgren Lawrence grading (right knee/left knee)(number [percentage])				
Grade 1	2 (4.2%)/2(4.2%)	NA	NA	NA
Grade 2	14 (29.8%)/16 (34.0%)	NA	NA	NA
Grade 3	24 (51.1%)/22 (46.9%)	NA	NA	NA
Grade 4	7 (14.9%)/7 (14.9%)	NA	NA	NA

BMI, body mass index; WOMAC, Western Ontario and McMaster Universities Osteoarthritis Index; HAQ score, Health Assessment Questionnaire score; n, number of patients or subjects; X^2^, value of chi-square test; t, value of Student’s t-test; NA, not applicable.

† P value for Fisher Exact test

* P is significant at <0.05

Genotype assessment of the patients and control subjects are illustrated in *Table 2*. The most common GDF5 genotype among patient group was the CT genotype, as was present in 23 patients (48.9%); TT was the most common in controls, as it was present in 16 subjects (40%). There was no statistically significant difference between the frequencies of different GDF5 genotypes (TT, CT and CC) between the patient group and control group (X^2^=2.441, *P*=0.3). Observed genotypes were tested and found to be consistent with Hardy-Weinberg (X^2^=2.324, *P*=0.127).

**Table 2.** Distribution of different Growth Differentiation Factor 5 genotypes according to the gender in patients and control groups.

**Group**	**Gender**	**GDF5 genotypes**			**X^2^**	***P***
		**TT**	**CT**	**CC**		
**Patients group**		**(n = 14 patients)**	**(n = 23 patients)**	**(n = 10 patients)**		
	Male (number [percentage])	2 (14.3%)	4 (17.4%)	1 (10.0%)	0.338	1.000^†^
	Female (number [percentage])	12 (85.7%)	19 (82.6%)	9 (90.0%)		
**Control group**		**(n = 16 subjects)**	**(n = 13 subjects)**	**(n = 11 subjects)**		
	Male (number [percentage])	4 (25.0%)	4 (30.8%)	3 (27.3%)	0.256	1.000^†^
	Female (number [percentage])	12 (75.0%)	9 (69.2%)	8 (72.7%)		

GDF5, Growth Differentiation Factor 5; TT, one of GDF5 genotype that has two (T) alleles; CT, one of GDF5 genotype that has two (C,T) alleles; CC, one of GDF5 genotype that has two (C) alleles; n, number of patients or subjects; X^2^, value of chi-square test

† P value for Fisher Exact test

* P is significant at <0.05

There were no statistically significant differences between different GDF5 genotypes (TT, CT, CC) and BMI among the patient group and control group (*P*>0.05).

Regarding total WOMAC index, there was a statistically significant difference between different GDF5 genotypes (TT, CT, and CC) (*P*≤0.001). However, total WOMAC index was significantly higher among patients with TT genotype compared to patients with CT and CC genotypes. Also, total WOMAC index was significantly higher among patients with CT genotype compared to patients with CC genotype (*Table 3*). Pain and stiffness subscales of WOMAC index were significantly lower among patients with CC genotype in comparison to patients with TT and CT genotypes. However, there was no significant difference between patients with TT and CT genotypes regarding pain and stiffness subscales (*Table 3*). As regards the function difficulty subscale, there was a statistically significant difference between different GDF5 genotypes (TT, CT, and CC). However, function difficulty was significantly higher among patients with TT genotype in comparison to patients with CT and CC genotypes; also, the function difficulty was significantly higher among patients with CT genotype in comparison to patients with CC genotype (*Table 3*).

**Table 3.** Relation between different Growth Differentiation Factor 5 genotypes with Western Ontario and McMaster Universities Osteoarthritis Index and Health Assessment Questionnaire among patients group.

**Assessment scores**	**GDF5 genotypes**			**H**	***P***
	**TT (n = 14)**	**CT (n = 23)**	**CC (n = 10)**		
WOMAC					
Mean ± SD	56.50±12.06^‡^	47.78±11.81^§^	35.0±8.31	15.588^*^	<0.001^*^
Median (Min.–Max.)	60.0(29.0–74.0)	48.0(22.0–73.0)	35.5(24.0–48.0)		
Pain subscale					
Mean ± SD	10.50±2.28^‡^	10.13±3.05^§^	7.50±2.01	8.733^*^	0.013^*^
Median (Min.–Max.)	11.0(5.0–15.0)	10.0(5.0–15.0)	7.5(5.0–10.0)		
Stiffness subscale					
Mean ± SD	3.21±1.63^‡^	2.26±1.45^§^	1.10±0.57	11.445^*^	0.003^*^
Median (Min.–Max.)	3.0(0.0–5.0)	2.0(0.0–5.0)	1.0(0–2.0)		
Function difficulty subscale					
Mean ± SD	42.79±9.0^†‡^	35.39±8.08^§^	26.40±6.92	15.863^*^	<0.001^*^
Median (Min.–Max.)	45.5(24.0–54.0)	34.0(17.0–53.0)	27.0(16.0–36.0)		
HAQ score					
Mean ± SD	1.16±0.23^‡^	0.97±0.30^§^	0.69±0.20	14.124^*^	0.001^*^
Median (Min.–Max.)	1.23(0.65–1.50)	1.10(0.50–1.50)	0.60(0.40–1.0)		

WOMAC, Western Ontario and McMaster Universities Osteoarthritis Index; HAQ score, Health Assessment Questionnaire score; n, number of patients; GDF5, Growth Differentiation Factor 5; TT, one of GDF5 genotype that has two (T) alleles; CT, one of GDF5 genotype that has two (C,T) alleles; CC, one of GDF5 genotype that has two (C) alleles; Kruskal Wallis test, analysis of variance test to determine the difference between more than two means.

† Significant difference (Post Hoc test Dunn’s) between patients with TT genotype and patients with CT genotype (P<0.05)

‡ Significant difference (Post Hoc test Dunn’s) between patients with TT genotype and patients with CC genotype (P<0.05)

§ Significant difference (Post Hoc test Dunn’s) between patients with CT genotype and patients with CC genotype (P<0.05)

* P is significant at <0.05

Regarding HAQ score, there was a statistically significant difference between different GDF5 genotypes (TT, CT, and CC) (*P*≤0.001). However, the HAQ score was significantly higher among patients with TT genotype in comparison to patients with CT and CC genotypes; also, the HAQ score was significantly higher among patients with CT genotype in comparison to patients with CC genotype (*Table 3*).

There was a statistically significant difference between different GDF5 genotypes (TT, CT, and CC) and K/L radiological grading of knee OA among the studied patients (*P*=0.029). It was observed that K/L radiological grade 2 was significantly more frequent among TT genotype (64.3%) compared to CT (26.1%) and CC (10%) genotypes. Also, K/L radiological grade 3 was more frequent among CT genotype (56.5%) in comparison to TT (28.6%) and CC (50%) genotypes. But, the frequency of K/L radiological grade 4 was significantly higher among CC genotype (40%) compared to CT genotype (8.7%) and TT genotype (7.1%) (*Table 4*).

**Table 4.** Relation between different Growth Differentiation Factor 5 genotypes with Kellgren Lawrence grading among patients group.

**Kellgren Lawrence grading**	**GDF5 genotypes**			**X^2^**	***P***
	**TT (n = 14) n(%)**	**CT (n = 23) n(%)**	**CC (n = 10) n(%)**		
**Right Knee**					
Grade 1	0(0%)	2 (8.7%)	0(0%)	8.630	0.134^†^
Grade 2	7 (50%)	5 (21.7%)	2(20%)		
Grade 3	6 (42.9%)	14(60.9%)	4(40%)		
Grade 4	1 (7.1%)	2(8.7%)	4(40%)		
**Left Knee**					
Grade 1	0(0%)^a^	2 (8.7%)^a^	0(0%)^a^	12.092	0.029^*†^
Grade 2	9 (64.3%)^a^	6 (26.1%)^b^	1(10%)^b^		
Grade 3	4 (28.6%)^a^	13 (56.5%)^a^	5(50%)^a^		
Grade 4	1(7.1%)^b^	2(8.7%)^b^	4(40%)^a^		

GDF5, Growth Differentiation Factor 5; TT, one of GDF5 genotype that has two (T) alleles; CT, one of GDF5 genotype that has two (C,T) alleles; CC, one of GDF5 genotype that has two (C) alleles; n(%), number and percentage of patients; X^2^, value of chi-square test.

† P value for Fisher Exact test

a,b Numbers with common letters are not significant (P>0.05) and numbers with different letters are significant (P<0.05)

* P is significant at <0.05

## DISCUSSION

The genetic background of primary knee OA involves multiple genes that encode proteins which have significant functions in the underlying disease process.^8^ The most important OA risk allele has been found to be rs143383, a C/T SNP located in the ′UTR of the GDF5 gene.^8^

The current study showed that there was no significant difference between OA patients and controls in the frequency distribution of the rs143383 SNP. This result is in accordance with the studies carried by Tsuzou et al.,^19^ Southam et al.,^20^ Cao et al.,^21^ and Shin et al.^22^

Tsuzou et al. reported the heterogeneous nature of OA genetic susceptibility. They reported that GDF5 (rs143383) SNP is not a risk factor for primary knee OA in Greek Caucasians.^19^ Southam et al. in Spain revealed no significant differences in genotype and allele frequency distribution of SNP in patients with knee OA and healthy control subjects.^20^ The same findings were reported from another two studies performed on the Korean population by Cao et al. and Shin et al.^21,22^

However, the results of the current study were not in accordance with the results of the studies conducted by Miyamoto et al.^23^ on Han and Japanese populations, Tawonsawtruk et al.^24^ on the Thai population, Mishara et al.^25^ on the North Indian population and Ozcan et al. on the Turkish population^26^ which revealed an association between GDF5 (rs143383) SNP and the risk of development of primary knee OA.

The difference and the discrepancy between the results of the current study and these studies reported worldwide might be attributed to several factors.

Egli et al. detected a second GDF5 polymorphism in the 5′ UTR region (rs143384) that could influence the expression of GDF5 rs143383.^27^ In addition, they identified a new polymorphism 2250ct that influenced the GDF5 allelic expression, independent of rs143383. The 2250ct polymorphism effect on the GDF5 allelic expression was corresponding to that seen for rs143383. In which, there is a moderate relative reduction in the expression on the order of 20%–25%.^27^

Furthermore, Reynard et al. reported that genetic effect of the rs143383 SNP was under the regulation of DNA methylation.^28^ They reported that DNA methylation regulates GDF5 expression in cartilage and modulates the functional effect of the OA SNP (rs143383).^28^ This is through interference with the binding of transcriptional repressor proteins which bind to and differentially repress the transcription of the two alleles of rs143383. This leads to allelic imbalance of rs143383 as observed in joint tissues obtained from patients with primary OA.^29^

In addition, ethnic differences in GDF5 methylation was reported,^29^ which is the result of differences in environmental and genetic factors. This could account for the differences in the results of different studies performed on GDF5 (rs143383) SNP in different populations.^30^

Finally, variation in the laboratory methods and techniques used by the genetic association studies might be another factor leads to inconsistent results in different studies.^31^ Furthermore, the current study used the WOMAC index and HAQ score to assess the genetic influence of GDF5 (rs143383) SNP on the severity and disability in primary knee OA patients. Regarding the WO MAC index, the patients carrying the TT genotype had the highest mean of total WOMAC index when compared to patients carrying the CC and CT genotypes. Also, function difficulty subscale of WOMAC index was significantly higher in patients with TT genotype in comparison to CT and CC genotypes. However, pain and stiffness subscales of WOMAC index were significantly lower among patients with CC genotype in comparison to patients with TT and CT genotypes, but there was no significant difference between patients with TT and CT genotypes regarding pain and stiffness subscales. Srivastava et al. reported that GDF5 was significantly associated with WOMAC-pain (*P*<0.001).^32^ Similarly, the HAQ score was significantly higher among patients with TT genotype when compared to patients with CT and CC genotypes (*P*<0.001). This indicates that GDF5 TT genotype is associated with more severe and disabling primary knee OA than the CT and CC genotypes.

Regarding the radiological assessment of knee OA severity, the current study showed statistically significant association between different GDF5 genotypes and K/L radiological grading of knee OA among the studied patients. K/L radiological grade 2 was significantly more frequent among TT genotype (64.3%) while K/L radiological grade 3 was more frequent among CT genotype group (56.5%), but the frequency of K/L radiological grade 4 was significantly higher among CC genotype (40%). This means that more knee OA radiological damage is associated with CT genotype and CC genotype. These results were in accordance with the study carried by Minafra et al.^33^ and Valdes et al.^34^ The Minafra et al. study on Sicilian primary knee OA patients reported a statistically significant association between genotype and K/L radiological grade for the GDF5 (*P*=0.02).^33^ Valdes et al. reported a significant association between tibiofemoral K/L grade and rs143383 SNP in patients with OA in UK population.^34^

The relation between rs143383 polymorphism and the severity of knee OA could be explained by the study conducted by Miyamoto et al.^23^ They reported that this SNP influences transcriptional activity in the core promoter of the GDF5 gene, with the T allele showed reduced transcriptional activity of GDF5 in chondrogenic cells. These findings suggest that rs143383 SNP may influence the biological processes that are involved in joint damage that might be caused by its relation with the progression of the disease.^23^

## LIMITATIONS

The current study had some limitations, which includes the following: the first limitation is the relatively limited number of patients and control subjects included in this study. This heralds the generalization of the results of the current study. Further studies on a larger scale are recommended.^35,36^

## CONCLUSION

In conclusion, the results of the current study revealed a possible genetic association between GDF5 (rs143383) SNP and severity of primary knee OA, which might facilitate the detection of patients with high risk for disease progression. The present study did not detect an association between the SNP and development of primary knee OA.
